# Usability and feasibility analysis of an mHealth-tool for supporting physical activity in people with heart failure

**DOI:** 10.1186/s12911-024-02452-z

**Published:** 2024-02-12

**Authors:** Andreas Blomqvist, Maria Bäck, Leonie Klompstra, Anna Strömberg, Tiny Jaarsma

**Affiliations:** 1 Department of Health, Medicine and Caring Sciences, Linköping University, Linköping, Sweden; 2 Department of Occupational Therapy and Physiotherapy, Sahlgrenska University Hospital, Gothenburg, Sweden

**Keywords:** mHealth, Heart failure, Physical activity, Disease management, Development, Co-design

## Abstract

**Background:**

Physical inactivity and a sedentary lifestyle are common among people with heart failure (HF), which may lead to worse prognosis. On an already existing mHealth platform, we developed a novel tool called the Activity coach, aimed at increasing physical activity. The aim of this study was to evaluate the usability of the Activity coach and assess feasibility of outcome measures for a future efficacy trial.

**Methods:**

A mixed-methods design was used to collect data. People with a HF diagnosis were recruited to use the Activity coach for four weeks. The Activity coach educates the user about physical activity, provides means of registering daily physical activity and helps the user to set goals for the next week. The usability was assessed by analysing system user logs for adherence, reported technical issues and by interviews about user experiences. Outcome measures assessed for feasibility were objective physical activity as measured by an accelerometer, and subjective goal attainment. Progression criteria for the usability assessment and for the proposed outcomes, were described prospectively.

**Results:**

Ten people with HF were recruited, aged 56 to 78 with median age 72. Data from nine of the ten study participants were included in the analyses. Usability: The Activity coach was used 61% of the time and during the first week two study participants called to seek technical support. The Activity coach was found to be intuitive and easy to use by all study participants. An increased motivation to be more physically active was reported by six of the nine study participants. However, in spite of feeling motivated, four reported that their habits or behaviours had not been affected by the Activity coach. Feasibility: Data was successfully stored in the deployed hardware as intended and the accelerometers were used enough, for the data to be analysable. One finding was that the subjective outcome goal attainment, was challenging to collect. A proposed mitigator for this is to use pre-defined goals in future studies, as opposed to having the study participants be completely free to formulate the goals themselves.

**Conclusions:**

It was confirmed that the Activity coach was easy to use. Furthermore, it might stimulate increased physical activity in a population of people with HF, who are physically inactive. The outcomes investigated seem feasible to include in a future efficacy trial.

**Trial registration:**

ClinicalTrials.gov identifier: NCT05235763. Date of first registration: 11/02/2022.

**Supplementary Information:**

The online version contains supplementary material available at 10.1186/s12911-024-02452-z.

## Introduction

### Background

Medical health practice using mobile devices aimed at the end user, is often referred to as mHealth, and such tools are predicted to have an important role to play in managing disease [1]. One area where mHealth could add value is in the management of heart failure (HF), a chronic and debilitating condition affecting more than 35 million people worldwide [2]. HF is a clinical syndrome characterized by breathlessness, peripheral oedema and fatigue as the most common symptoms [3]. People with HF are advised to be physically active [3], but in spite of physical activity being associated with improved prognosis [4], a majority of people with HF display low levels of physical activity [5]. Barriers to adherence to physical activity for this population, like low motivation, fear of worsening symptoms and lack of influence over what activities to perform [6], could potentially be addressed by a home-based mHealth-tool. We sought to develop a tool to support physical activity, based on the Medical Research Council (MRC) guidelines on developing and evaluating complex interventions [7]. These guidelines emphasize co-design, identification of stakeholders on all socio-ecological levels and advocate the use of a mixed-methods approach [7–9].

While modern technology is important in realising positive behaviour modifications [10], a tool is only efficacious if it meets the needs of the user [11]. To avoid the often-high rates of attrition of 50–80% [12], focus on usability is key. Usability is defined as the extent to which a product can be used by specified users to achieve specified goals with effectiveness, efficiency and satisfaction in a specified context of use, or the ease of use and acceptability of a system or product for a particular class of users carrying out specific tasks in a specific environment [13].

**Fig. 1:**
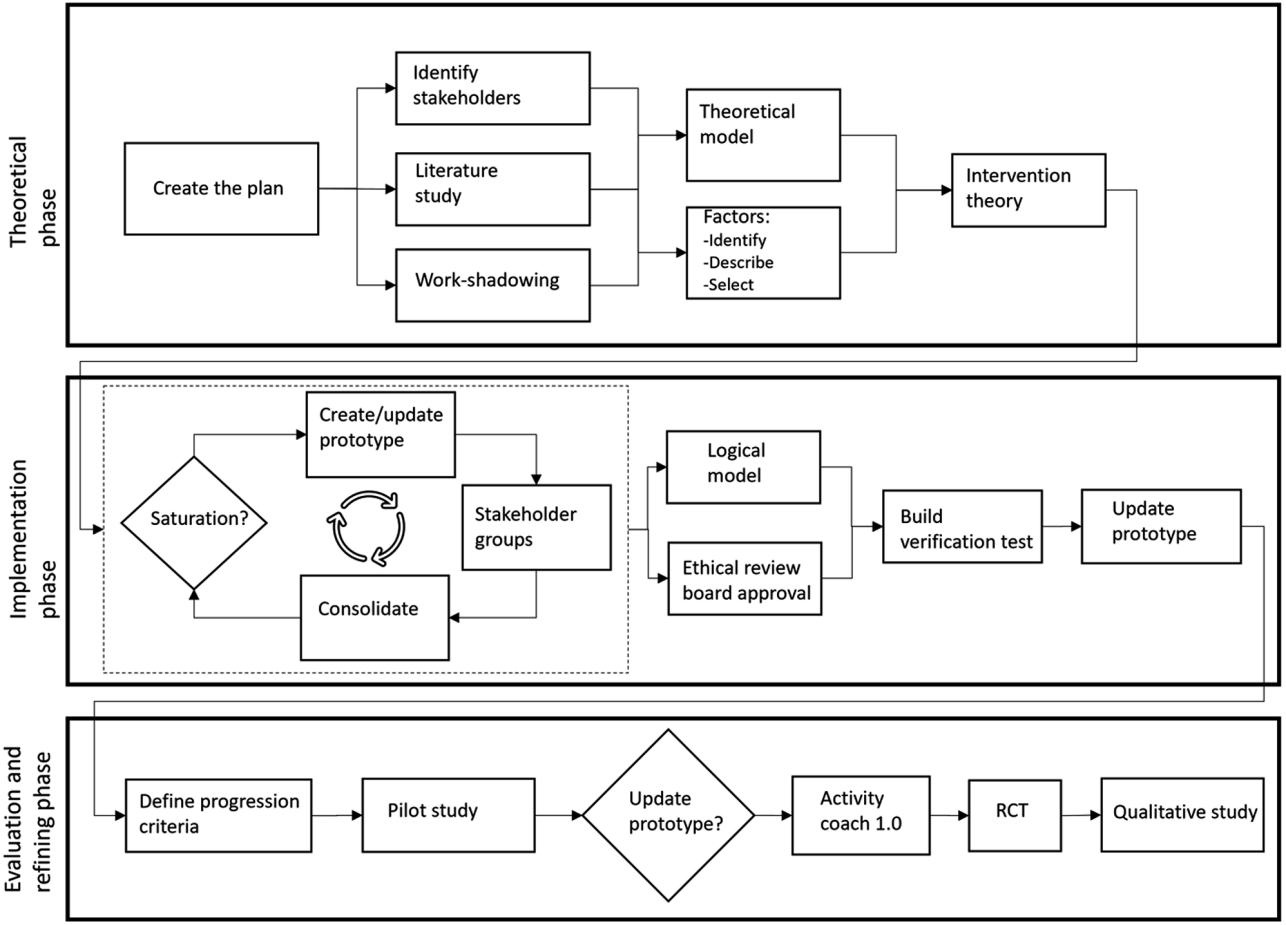
The development process of the intervention. The theoretical phase ensures a theoretical basis for the intervention is established. In the implementation phase the key-output is a working prototype. The prototype is then tested and refined in the final phase. RCT– randomized controlled trial

The development process used was based on the MRC guidelines [7], the INDEX (IdentifyiNg and assessing different approaches to DEveloping compleX interventions) guidance [14], and the 6SQuID (six essential Steps for Quality Intervention Development) model [15]. These different guides were combined to create a pragmatic process, which did not take too long to complete and the process is illustrated in Fig. 1. The first two phases of the development process are described in detail in the Appendix A “Development project: Activity coach”, and in the main section of this paper we focus on the third phase, the “Evaluation- and refining phase”. The tool developed will be referred to as the “Activity coach” throughout this paper.

### Aim

The aim of this study was to evaluate the usability of the Activity coach and to assess feasibility of outcome measures, for people with HF.

## Methods

### Design

To address the aims of this study, a mixed-methods design was employed. Usability was assessed through analysis of system adherence, technical issues reported by the study participants, as well as through probing of user experiences. To assess feasibility of outcome measures, we defined progression criteria for the outcomes tested. Data was collected from people with HF, who were recruited to use the Activity coach for four weeks (28 days). Former research show that 8–10 people usually is enough to identify about 80% of the usability issues [16]. The ethical review board approval for this research was obtained 2020-05-11 (ID: Dnr 2020–01444, Etikprövningsmyndigheten, Box 2110, 75,002 Uppsala, etikprovning.se). The study is registered at clinicaltrials.gov with study identifier NCT05235763.

### Intervention

#### The existing tool

The Activity coach was integrated on an existing home-based tool called Optilogg, developed for use in HF care [17]. Optilogg is a tablet computer wirelessly connected to a weight scale and incorporates symptom monitoring, interactive education with information for people with HF, and a flexible loop-diuretics regimen. Trends of data can be shared with the health care provider during a visit, at the discretion of the person using the tool. The user is encouraged to use Optilogg daily to register weight, get today´s dose of loop-diuretics and brief education about living with HF, and every five days the user is asked to assess his/her symptoms. Optilogg has been shown to enhance self-care behaviour and achieve a high system adherence in the elderly HF population [18, 19].

#### The activity coach

Theories of behaviour change can provide guidance to developing interventions aimed at sustained change in health behaviours, and such research indicates that to maintain new behaviours, there needs to be self-regulation skills and abilities, but also the resources required to perform the desired activities [20]. It has been proposed that antecedents of self-regulation skills are three personal perceptions. These personal perceptions need to be established before a new behaviour can manifest [21], and these are:

1. “positive outcome expectancy”, that the person must believe that the behaviour in question indeed has the effects communicated to the person.
2. “self-efficacy”, the person must believe that he or she can carry out the behaviour or activity.
3. “goal congruence”, the person must also feel that the outcomes of the activity are desirable and worth the effort.

In the design of the Activity coach (see Appendix A), the establishing of these three perceptions was deemed necessary, before any actual motivation to be physically active could appear. Furthermore, the Activity coach needed to be feasible, which means it can be used by an individual as part of their daily routine [11].

The Activity Coach starts with a three-step education during the first week, where positive outcome expectancy, self-efficacy and goal-congruence is hopefully achieved. After that initial week the patient is expected to be motivated to engage in physical activity and a short slideshow on the screen of the Activity coach illustrates how the person using the Activity coach can manually register physical activity. According to the previously mentioned design principle, the Activity coach needed to be an easy part of daily routine, so the registering of physical activity was made very quick and easy, but thereby also sacrificing opportunities to increase granularity of the reporting, i.e. by specifying intensity or type of activity registered. Trends of the registered activity can be viewed on the screen of the tablet. Furthermore, at the end of every week the user will receive a weekly summary on screen with the option to set a goal for next week. The goal functionality is optional so that anyone who feels negative stress from the goal, can simply select to not have a goal.

A more in-depth description of the Activity coach, the underlying research and the different features and parts of the Activity coach, as well as screen shots, are available in the Appendix A.

### Study participants

The inclusion criteria were age ≥ 18, confirmed heart failure diagnosis with functional class NYHA II-III, and signed written informed consent. Candidates who were currently enrolled in another study involving physical activity were excluded. The study participants were recruited from a primary care health centre in Stockholm, Sweden by their treating physician, randomly selected from listed people with a confirmed HF diagnosis. Those who accepted to participate in the study were contacted via telephone by the researcher. A letter was sent to all study participants including some more information and the consent form. At a scheduled home visit, the consent forms were collected, and the Activity coach was installed, and the study participants were shown how to use the Activity coach. During this visit, the researcher asked the study participant to define the two goals for the goal attainment outcome. The hardware required to use Optilogg with the Activity coach installed, i.e. a 7” tablet and a connected weight scale, were supplied to the study participants for the duration of the study.

### Data collection and analysis

#### Usability of the activity coach

##### System adherence

System adherence was defined as the number of days that the study participant self-reported physical activity in the Activity coach, divided by the length of the study (i.e., 28 days) and will be reported as a percentage, i.e. corresponding to the frequency of use.

##### Technical issues

All study participants were provided a telephone number that they could call during office hours for technical support with the Activity coach. All calls were registered continuously throughout the study.

##### User experiences

Semi-structured interviews were conducted by the first author at the completion of the study, i.e. after four weeks. The interviews were not recorded, but instead documented using field-notes, which were digitized immediately after each interview (the interview guide is available in the Appendix B). The field-notes were analysed using qualitative content analysis, with an inductive approach [22]. Meaningful units were selected from the notes and subject to open coding, where sentences or messages were classified into much smaller content headings (or codes). This procedure was done twice and by two different people in the development team, to update and refine the headings. Next, the headings were grouped into categories. The final step of this analysis is abstraction, where a general description of the findings is formulated based on the identified categories [22].

#### Feasibility of outcome measures

##### Objectively measured physical activity

Suggested preliminary outcome measures include steps per day, as well as reduction of sedentary time by ≥ 10 min per day. Interruptions of sedentary behaviour was analysed by average number of sedentary breaks per day. These outcomes are based on objective physical activity, measured with an accelerometer.

##### Subjective goal attainment

Goal-attainment is a suitable outcome measure for populations with heterogeneous disease stages [23]. The researcher and the study participant define two goals relating to increased physical activity and each goal is accompanied by five possible levels of achievement from bad to good on an ordinal scale (-2, -1, 0, 1 and 2), where zero corresponds to no change. The mean value of the two goals at follow-up is the statistic to analyse, but the goal scores are also reported individually.

#### Accelerometer data

The study participants were given an ActiGraph GT9X (Pensacola, FL, USA), a valid and reliable accelerometer [24], and instructed to wear it daily for a week, removing it only for showers and while asleep. The accelerometer was to be worn around the hip with an elastic belt. After that week the accelerometers were mailed to the researcher so that the data could be retrieved. The accelerometers were then delivered to the study participants again for them to use during the fourth week. All data recorded in the accelerometers were analysed using the ActiLife software (version 6.13.4), calculating the number of steps per day and sedentary time using Copeland’s cut-points for older adults [25]. Based on the accelerometer counts per minute, these cut-points were 0–99 = sedentary, 100–1039 = light physical activity and ≥ 1040 = moderate to vigorous physical activity.

#### Final data collection

At the end of the four-week study, the accelerometer and Activity coach hardware were retrieved, the previously selected goals were evaluated, and the semi-structured interviews were conducted. Goals were graded on the scale from − 2 to 2 by the study participant. The field-notes from the interviews were immediately manually transferred to a digital format for further analysis as described earlier.

### Progression criteria

We used an approach inspired by Hawkins et al. [26] to predefine progression criteria for the measures of usability and feasibility. In a previous study of a home-based eHealth intervention, a system adherence ≥ 60% was used as a cut-off point to describe adherence [27]. We also defined acceptable levels of accelerometer use [28]. The progression criteria are described in Table 1.

**Table 1:** Progression criteria for the interventions and planned outcomes

Type	Progression criterion	Measures used	Assessment of whether criterion has been met
Usability	Patients can successfully use the activity module.	System adherence. Technical support required.	Activity coach adherence$\ge$ 60% (median of population) After the first week of use, < 20% of study participants need to contact technical support
	The activity module features work as intended.	User feedback from interviews.	The result from the interviews was that the information material in the Activity coach was deemed relevant. The goal-setting function did not cause negative stress in > 20% of patients.
Feasibility of outcomes	Accelerometer is used enough to constitute data-source for primary outcome.	Data stored in the accelerometers.	> 80% of study participants use the accelerometer for ≥ 4 days/week, with a minimum wear time of 540 min/day.
	Goal-attainment outcome feasible.	At baseline, each study participant will together with the researcher define goals to achieve.	It was feasible with a reasonable amount of effort to define two goals per patient. Goals were connected to the implemented mechanisms of change.

## Results

### Study population

Thirteen people with HF were asked and consented to participate in the study, but three withdrew their consent prior to starting the pilot. One had been admitted to the hospital and the two others did not state any reason. Ten people with HF had the Activity coach installed. The median age was 72 (quartile range 65–76) and seven were male. Half-way through week one, one study participant chose to withdraw from the study and was therefore not included in the analyses. No reason for this withdrawal was given.

### Usability of the activity coach

#### System adherence

The median Activity coach adherence was 61%, IQR 34-78%. Three study participants did not register physical activity at any point during the study and the maximum adherence was 91%. The progression criterion was met.

#### Technical issues

During the first week of the intervention, two study participants called the tech-support number for assistance, and one called to state the desire to exit the study. After those three calls, no further tech-support calls were registered. All deployed systems had successfully recorded the data as intended and the data could be retrieved, processed, and analysed according to expectations. The progression criterion was met.

#### User experiences

The nine study participants who completed the entire pilot were interviewed at the follow-up and the interviews ranged between 15 and 35 min, with a median time of 28 min. Analysis of the data by two independent reviewers resulted in 45 codes across the nine study participants who were interviewed. These were grouped into three categories, which were subsequently listed in order of importance (based on frequency of occurrence). These were “ease of use”, “motivation to be physically active” and “increased knowledge and valuable information”. The abstracted descriptions of these categories are described below.

##### Ease of use

All of the study participants felt comfortable with the Activity coach after using it for a while, in spite of some of them feeling worried at first. All study participants reported that it became a habit and was easy to use the tool.

First, I thought to myself, will I be able to do this? And then it was easy and became a habit. #12, woman, 68.

It just improved over time. It was easy to enter data. #4, man, 76.

##### Motivation to be physically active

There was a general feeling about being more motivated to be physically active. Some quotes relating to motivation, were

It gives you a little kick in the butt, to get going. #13, man, 75.

and

I believe others can also be motivated by this; you see the results so quickly. #2, man, 78.

Other comments were relating to an increased sense of awareness about physical activity, like a continuing little nudge or mental focus on physical activity, which resulted in motivation to perform the activity. There was also an opinion voiced that the Activity coach maybe didn’t push you enough:

I have lived my life like I always do. My wife said that [the Activity coach] should have pushed me more, been on my tail so to speak. #4, man, 76.

##### Increased knowledge and valuable information

Many participants claimed to have a high level of knowledge about physical activity prior to the intervention and claimed to already know most of the content, however several described the information as valuable and that they increased their knowledge. This increased knowledge did not, however, always lead to any action.

The information I read, I more or less knew already. #3, man, 77.

One quote to illustrate how others indeed learned from the intervention, but claim that it didn’t translate into actions:

I have absolutely learned new things, but I haven’t changed any habits like I should have. #4, man, 76.

There were more signs of knowledge uptake. One participant said the following about this:

I have increased my knowledge about life-style aspects and physical activity […] and that it isn’t dangerous for me to get short of breath or muscular fatigue. #8, woman, 56.

To summarize the qualitative analysis, the participants who used the Activity coach perceived it as easy to use, despite initial concern about novel technology in some cases. It also appears that the Activity coach leads to an increased awareness about physical activity and several study participants learned new things from the intervention.

### Feasibility of outcome measures

#### Objectively measured physical activity

Out of the nine study participants who participated in the whole study, eight (89%) of them used the accelerometer ≥ 540 min a day and on average the study participants used the accelerometer 100% of the days they were asked to wear it. The wear time per day was on average 707 ± 123 min and the average number of days the accelerometer was worn was 7.4 ± 0.7. The progression criteria for accelerometer-based outcomes were met (Table 1).

**Fig. 2:**
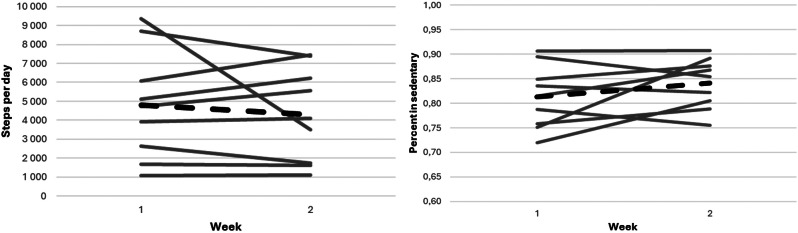
The panel to the left shows the average steps per day for the study participants and how it changed from week 1 to week 4. The panel to the right shows the percent of time spent in a sedentary state and how it changed from week 1 to week 4. The dashed lines represent the population average

The average number of steps per day was 4 350 week 1 and 4 300 week 4. The time spent in a sedentary state was 83% week 1 and 84% week 4. Three study participants decreased their sedentary time by more than ten minutes, and six increased their sedentary time. This is illustrated in Fig. 2.

For week 1, there was on average 104 ± 23 sedentary breaks, and for week 4 that number was 105 ± 21.

#### Subjective goal attainment

Most of the study participants could provide two goals, relating to the implemented mechanisms of change. It was however not possible for each study participant to formulate goals, and there was a tendency to be vague in describing their goals, e.g., “feel better”, making it harder to rate or score the level of goal attainment at the follow-up.

Eight of the study participants could score their personal goals, and one of them only stated one goal to begin with. The mean value of the two goals combined was 0.19. Two reported improvements, one reported deterioration and the remaining five reported no difference. When analysed separately, the mean value of the first goal was 0.38 and 0 for the second goal.

The progression criterion was not clearly met.

## Discussion

This study investigated the usability of the Activity coach, using a mixed-methods design. We also assessed feasibility of outcome measures, which could be used to study potential efficacy of the Activity coach in future research.

All study participants found the Activity coach easy to use and a majority felt motivated to increase their physical activity, which is the end goal of using the Activity coach. We observed that several study participants claimed early on in the interviews that they didn’t perceive any benefits of having used the Activity coach, but as the interview progressed it was very common that the respondent came to the conclusion that motivation to be more physically active in fact was higher than before. It was a recurring theme in the interviews that the respondent believed the Activity coach would be better suited for a sicker, less active person. Future work should explore the most suitable patient group to use the tool, for example if the tool would be more effective in patients that are more inactive. If that is to be pursued, a means of screening for physical inactivity is needed. In previous research, it was shown that a single-item self-report question might suffice to identify an appropriate study population [29]. In that particular case, the question used was item nine from the European Heart Failure Self-care Behaviour Scale, i.e. “I exercise regularly”, which is answered on a five-level ordinal scale [30].

The frequency of inputting physical activity was close to the pre-specified lowest acceptable limit of 60%, so it might be appropriate to investigate if some improvements in terms of usability can be made before proceeding with further studies of the Activity coach. The lower-than-expected adherence might be because the study participants were overwhelmed with the other features of the Optilogg and it might be appropriate to have people with HF first getting used to the standard Optilogg, and only then introduce the Activity coach. Other studies have also reported an initial threshold to “get started” with an mHealth intervention, similar to what we found [31–33]. The Activity coach, however, entails more than only the self-monitoring of physical activity, such as the education about physical activity, visual feedback and personal goals. This implies that a user could potentially interact and use the Activity coach in ways not captured in this metric, and we may as a consequence underestimate the adherence.

The fidelity of data recorded in the Activity coach, as well as with the accelerometers was sufficiently high and suitable for use as outcome measures.

The personal goal setting proved more challenging than anticipated. Some study participants defined either vague or no goals, resulting in difficulties scoring the goal attainment at follow-up. One mitigator could be to use a list of pre-defined goals for the study participants to choose from [34], and another approach might be to set the goals together with a health care professional.

### Limitations

Only field-notes were used, rather than recorded interviews, in spite of recordings and subsequent verbatim transcripts providing more reliable data [35]. Although impressions, emotions, and contextual details are better recorded through field-notes since ideas and memories from interviews are likely to be lost further down in the research process [36], these benefits of field-notes should not preclude recording the interviews as well as taking the field-notes.

Three out of the original thirteen patients chose to withdraw their consent prior to even starting the study, and one immediately after the study had started. This could indicate a fear of technology, that might lead to selection bias in a larger study, and selection bias is common in studies of mHealth interventions [37, 38] and could lead to issues concerning external validity of reported findings.

## Conclusions

The Activity coach was easy to use for people with heart failure. The Activity coach may stimulate increased physical activity in an appropriately selected population of physically inactive people with heart failure, but some improvements to the Activity coach should be made to ensure a higher adherence. The investigated outcomes are feasible to use.

### Electronic supplementary material

Below is the link to the electronic supplementary material.

Appendix A – Development project: Activity coach

Appendix B – Interview Guide

## Data Availability

The datasets used and/or analysed during the current study are available from the corresponding author on reasonable request. Please consult appendices first.

## Abbreviations

HF: heart failure
MRC: Medical Research Council
6SQuID: six essential Steps for Quality Intervention Development
RCT: Randomized Controlled Trial
