# Supplementary material for: Usability and feasibility analysis of an mHealth-tool for supporting physical activity in people with heart failure
Source: BMC Med Inform Decis Mak. 2024 Feb 12;24:44. doi: 10.1186/s12911-024-02452-z (PMC10860324; doi:10.1186/s12911-024-02452-z)
Supplement: Supplementary file 1 — Appendix A – Development project: Activity coach [file 12911_2024_2452_MOESM1_ESM.docx]

Appendix A

Development project: Activity coach

# Introduction

This appendix describes the development process from the first steps including defining the development plan up to when there was a fully functioning prototype ready for testing in a pilot study, which is reported in the main article.

# Methods and materials

The development plan was primarily based on the MRC guidelines (1) and the INDEX guidance (2), but also incorporating steps one through three of the 6SQuID model as means of defining an intervention theory (3). However, even though the 6SQuID model is pragmatic, other meticulous development guidelines span over many years and mHealth is a rapidly changing field (4). To reduce the time required for development, we also incorporated input from published papers specifically written on development of mHealth (5-7). The development plan is illustrated in Figure A1.


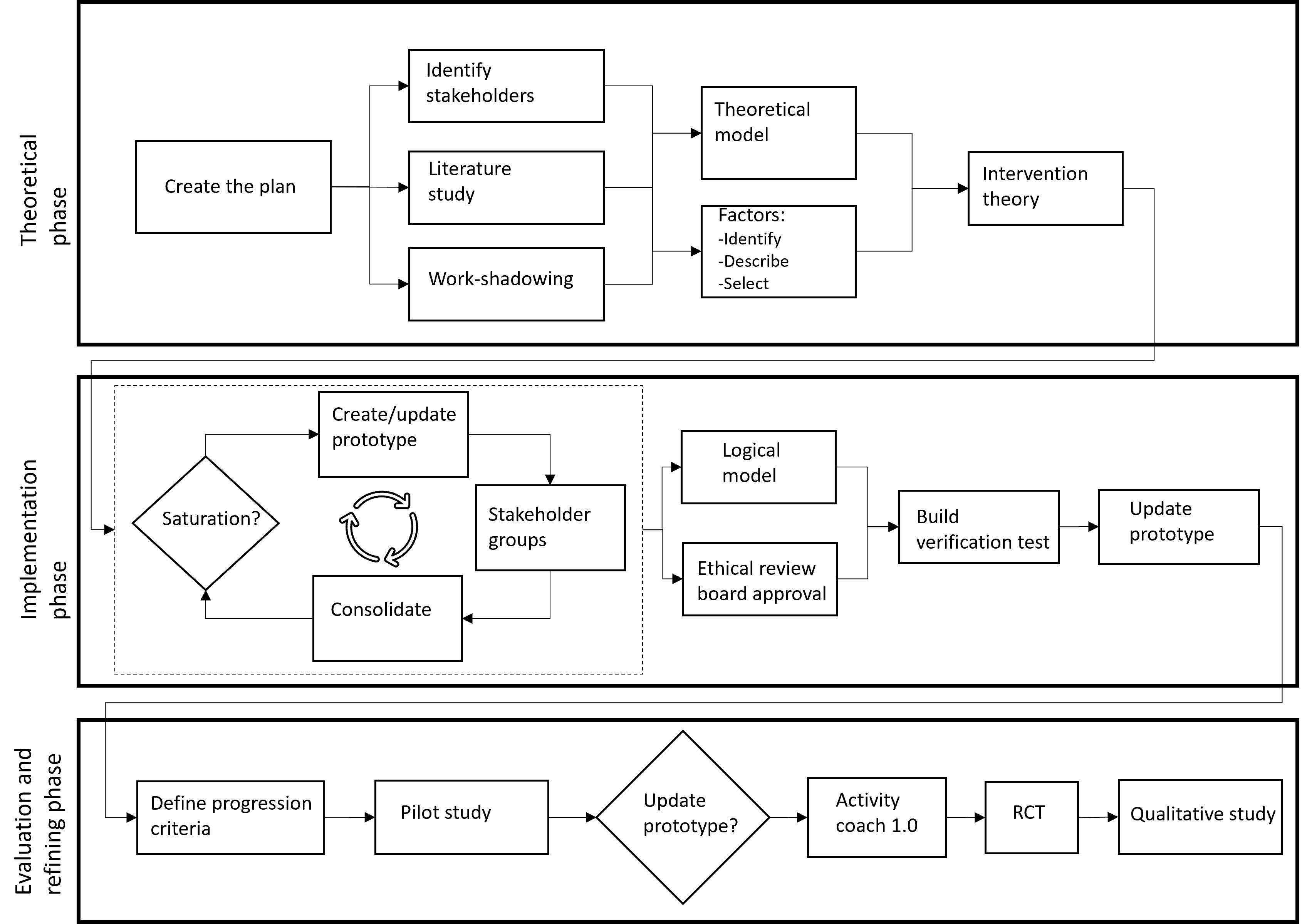


Figure A1. Illustration of the development process. RCT – randomized controlled trial.

In the theoretical phase the main outcome is an intervention theory, which is a description of how to deliver the change mechanism. For complex interventions it is important to not use off-the-shelf theories, as they often are too narrow for a complex situation, (8). We therefore first defined a theoretical model to be used as the theoretical framework for the intervention. In parallel we identified problems worth solving, here labelled factors. In accordance with the 6SQuID model, we then described the cause and effect of identified factors, and finally selected the factors which are both malleable and impactful. The key-outcome of the theoretical phase is the intervention theory which will serve as directions on to the tool to develop. The second phase is the implementation phase, where the theoretical intervention was turned into a tool for patients. The implementation (or prototyping) was performed in an iterative manner, which is appropriate for developing behavioural interventions (8).

We wished to use co-production in this development project, so small groups of stakeholders were identified and invited to play an active role in the prototyping (9) In our project these groups were based on the stakeholder identification from the first phase. After a certain number of iterations, the development team concluded that some kind of saturation was achieved, in that only incremental value would be added with further iterations, at which point the iterative loop was exited and a logical model to describe the intervention could be drafted. The prototype was then ready for a build verification test (BVT) or “smoke testing”, where patients test it on the field, to identify bugs or erroneous behaviours that might persist. Before doing so, an ethical review board approval must be obtained, to allow for the BVT and the subsequent pilot test in the following and final phase. The purpose of the BVT was to identify technical issues and assess core functionality to ensure the prototype is stable and can be tested more thoroughly. It is hence not a purpose of the BVT to assess usability or efficacy. Following potential updates to the prototype after the BVT, a new version of the prototype is the key output of the implementation phase, and the evaluation and refining phase is initiated.

To assess usability we used a mixed-methods approach, as recommended by the MRC (1). Quantitative data was obtained from the tool itself as well as other data collected, and qualitative data collected by semi-structured interviews, carried out after the intervention, to evaluate and probe the user experiences. The interviews were analysed using qualitative content analysis, with an inductive approach (10).

# Results

## Theoretical phase

The key outcome of the theoretical phase is the intervention theory, which will be a product of the theoretical model and the identified factors. The stakeholders were identified as people with HF, HF nurses, cardiologists, and physiotherapists. A literature review was made and summarized into a guiding document and the first author work-shadowed a physiotherapist working with HF patients.

A theoretical model was constructed as an adaptation and synthesis of other models and theories in the fields of physical activity, behaviour change and heart failure, published in peer-reviewed journals (11-18), and is illustrated in Figure A2.


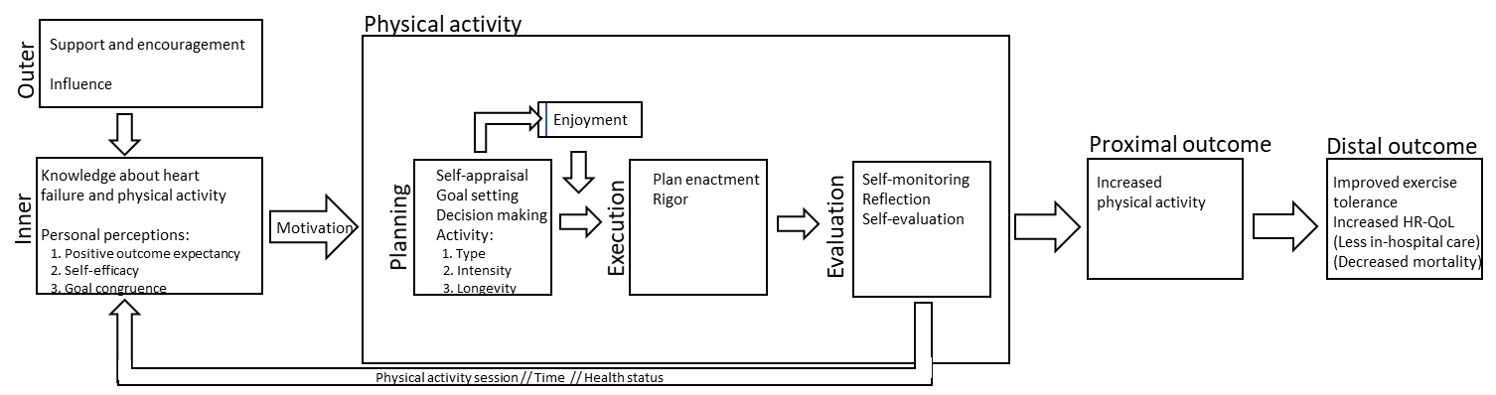


Figure A2. Theoretical model. The theoretical model for behaviour change relating to physical activity for people with heart failure.

The constructs of the model and associated concepts:

- Outer
  - *Support and encouragement* – typically from family, friends, or physical therapist.
  - *Influence* – media, health care professionals, family, and friends.

These outer concepts will influence the person’s knowledge and perceptions.

- Inner
  - *Knowledge about heart failure and physical activity* – factual knowledge about the disease, about physical activity, and the association between the two.
  - *Personal perceptions* – this concept has three constituents (or steps) and all need to be in place for motivation to occur. It is a personal journey through these three steps, which we describe like this:
    - 1 – Positive outcome expectation – a person has to internalize and fundamentally believe that the outcome of physical activity is positive, that it in fact leads to what has been taught it leads to.
    - 2 – Self-efficacy – There must be faith in one’s ability to perform the physical activity alluded to in step 1 above.
    - 3 – Goal congruence – The belief and opinion that the outcomes of the physical activity not only will occur (step 1) and that one can do them (step 2), but the outcome must also be a personally desirable outcome, that is in line with other desires and wishes, and also that the benefits outweigh the negative aspects.
- Physical activity
  - Consists of three constructs:
  - *Planning*
    - *Self-appraisal* – the ability to assess health status and allow that assessment to guide modification of the physical activity.
    - *Goal setting* – Defining goals, both in the short term like aspects regarding the activity to perform (e.g. length of the walk, a target number of repetitions, etc) and in the long-term, pertaining to what a person wants to achieve. Being able to set goals is important.
    - *Decision making* – Practical aspects, like setting aside time, purchasing equipment, prioritize.
    - *Activity* – concrete aspects of the physical activity, which activity, in what manner it is to be executed, how long/how many/how often/intensity, alone or in a group etc.
  - *Execution*
    - *Plan enactment* – the activity actually being performed.
    - *Rigor* – the intensity of the activity, or the passion with which it was carried out.
  - *Evaluation*
    - *Self-monitoring* – objectively tracking/registering and processing information about the activity performed.
    - *Reflection* – subjectively noting, internalizing, and processing information about the activity performed.
    - *Self-evaluation* – what went/felt well/good, what went/felt bad, what conclusions to bring to the next step.
- Proximal outcomes
  - The increase in physical activity is the primary outcome.
- Distal outcomes
  - Improved exercise tolerance.
  - Increased health related quality of life (HR-QoL).
  - Potentially fewer in-hospital days.
  - Potentially decreased risk of mortality.
- Mediator
  - When a person has internalized a certain causal link between physical activity and a certain outcome, is of the opinion that he/she can perform said activity and finally sees the aforementioned outcome as something desirable, this creates motivation to engage in the activity. We therefore view “Motivation” as a mediator between the constructs “Inner” (i.e., internal processes/states) and “Physical activity”.
- Moderator
  - That which will determine to what extent the physical activity is taking place, and with which rigor it will be performed, is the enjoyment or appreciation of the activity in question. We believe that “Enjoyment” moderates the link between the “Planning” and the “Execution”.

The physical activity taking place, will affect the “Inner”-construct and shape the person’s views and opinions on the topic. The “loop” illustrated in Figure A2 will be triggered either by an activity taking place (like a walk, or cleaning the house), or by time passing or the improvement or deterioration of health. These factors will in turn influence the “Inner”-construct.

The development team identified a list of factors according to the 6SQuID model (3) based on interviews and literature studies and the factors which were deemed to be the most impactful, but yet malleable were selected and are listed in Table A1.

**Table A1.** Description of the selected factors and their respective causes.

| **Factor** | **Cause** |
| --- | --- |
| Patients do not believe they should be physically active | Family advises the patient to rest and cautions them |
|  | Unclear advice on rest and activity |
| Patients do not believe they can be physically active | Poor self-efficacy can manifest in beliefs such as  “I am too old, weak or sick to be physically active” |
|  | Fear of movement: Movement causes symptoms, which is very unpleasant |
| Patients lose motivation and simply forget | Nothing is there to remind them when they are not with a health care professional |
| Patients are digitally isolated and not participating in connected life | Alternatives to social support and public facilities are readily available through the internet and smartphones, but not all patients use these channels (e.g., age and education dependent) |
|  | Health-care professionals might not prescribe novel technologies to non-tech savvy patients |

Following this identification and description, the factors (or certain aspect of a factor) having the greatest scope for change as described in literature and interviews, were re-formulated and these factors and the corresponding evidence for being modifiable are listed in Table A2.

**Table A2**.The factors most likely to be malleable and the underlying reasoning.

| **Factor** | **Action** | **Evidence-based claim** |
| --- | --- | --- |
| Limited motivation | Motivate through personalized goal-setting and tailored activities | Motivation can increase when the patient is allowed to choose the activity (18) |
| Patients’ personal beliefs and self-efficacy | Inform and educate the patient on physical activity in the context of HF | Education and interventions aimed at self-efficacy modification work (19) |
| Fear of movement due to symptoms | Inform and educate the patient on potential benefits of physical activity, and change negative beliefs | Fear of movement is related to negative beliefs about and understanding the necessity of physical activity. Tailored information is one important need. (20) |
| Lack of reminding, nudging and motivation at home. | Using modern technology (mHealth), create motivation and nudging at home, and include physical activity as a natural part of self-care | A home-based tool can positively affect self-care behaviour among patients with HF (21, 22) |
| Patients are not IT-savvy, and don’t get access to new tools | Create or use tool specifically developed for this population | The above-mentioned tool is designed for and validated on this population with a high system adherence. (21, 22) |

The proposed mechanisms of change based, is the foundation for our intervention theory and it was formulated as follows:

- Inform and educate the patient about physical activity and HF, with the proximal outcome to change beliefs, with the distal purpose of improving self-efficacy and motivation
- Track physical activity which is selected by the patient and allow for individual goal setting, to further increase motivation
- Give access to a home-based tool with reminders and information on activity and HF.
- Educate and stimulate health care providers to advise the use of the tool to patients

## Implementation phase

### Iterative process

With an intervention theory established, the next phase of the development plan is the implementation phase, in which the different stakeholder groups are engaged to define the actual design of the intervention and eventually lead to a prototype ready for testing. With the literature review, the theoretical model and the intervention theory as the foundation, the iterative process with the stakeholder groups was initiated. The first iteration started with two separate sessions with physiotherapists where the general scope of the intervention was discussed, as well as their unmet needs. Following those sessions, a first draft of the intervention which we called the “activity coach” was designed. Mock-up versions of the activity coach were presented to 11 people with HF. While they were observed, they gave verbal feedback on the graphical user interface (GUI) and functionality. This led to a new version, which was subsequently presented to a new group of people with HF the same way, with feedback inspiring a new version. Similar sessions took place with occupational therapists, physiotherapists, HF nurses and cardiologists, in the iterative manner illustrated in Figure A1. The activity coach gradually took form, and the iterations proceeded until consensus in the development team was that an appropriate level of maturity of the activity coach had been reached and no significant new input appeared, such that saturation was achieved.

Some of the key features and/or design aspects that the iteration-phase yielded are:

- The goal of the activity coach was to encourage every day physical activity, as opposed to exercise.
- The activity coach must be individualized due to the heterogeneous nature of the HF population.
- The activity coach should serve as a reminder, but also provide feedback on progress. These are two important factors to maintain a new habit.
- For motivation to be physically active to occur, three distinct perceptions must manifest: 1 – Positive outcome expectancy, 2 – Self-efficacy, and 3 – Goal congruence. This should be achieved through a physical activity-specific education module.
- Goal setting is important for most patients, to stay motivated and know what to strive for. At the same time, it needs to voluntary to set goals, as some patients perceive goal setting as stressful.
- Any interaction with the activity coach needs to fit the preferences of the user.

### Description of the prototype

The activity coach starts with an introduction to physical activity, and then during the first week educates the user with the purpose of achieving the personal perceptions in the theoretical model; positive outcome-expectancy, self-efficacy and goal-congruence, to the point where the user is motivated to engage in physical activity. At that point a slide show is shown on screen to explain to the user how to register physical activity, and how to check trends and past achievements. At the end of each week a weekly summary is shown to the user and there is an opportunity to set a goal for next week. Should the goal-setting functionality feel stressful to the user, it is possible to simply select “no goal”. The selected goal (if any) is illustrated in the History-tab, where the user can track the progress made.

-
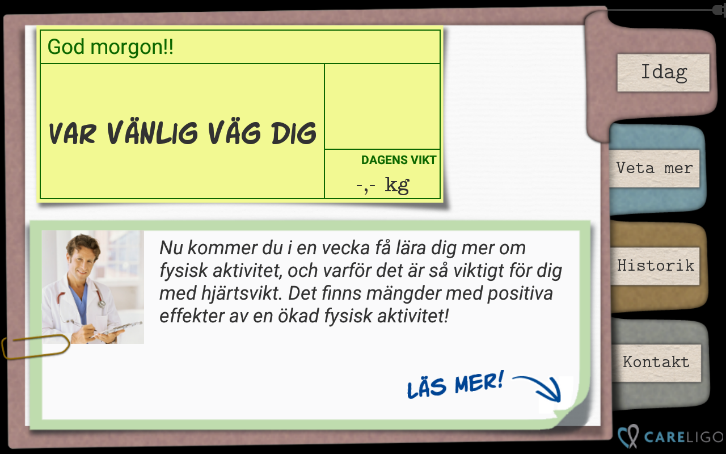
The first day that the activity coach is initiated, the frame of the short tip changes colour (indicated here by the blue circle) and informs the user that the upcoming daily tips will be about physical activity.

Figure A3. First day of intro week.

- The user can, if desired, read more about the importance of physical activity by pressing “READ MORE” (Swedish: ‘LÄS MER’).
-
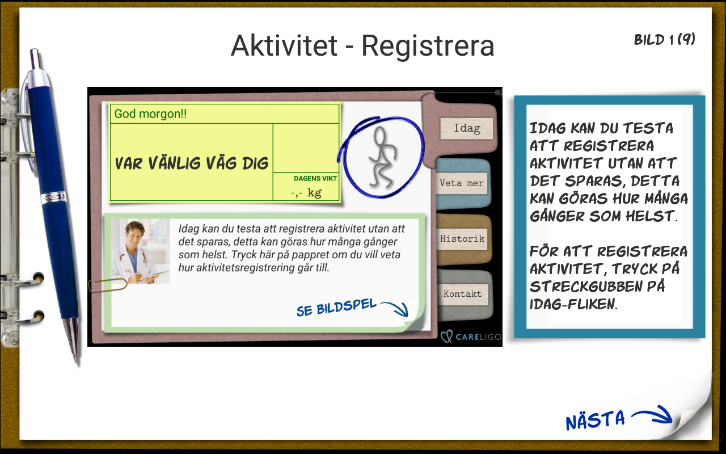
At the end of the first week, a slide show appears on screen (this is picture 1 of 9), with the purpose of informing the user how to register physical activity.

Figure A4. Last day of intro week.

- This first slide illustrates with a blue circle, a new symbol appearing on the Home-screen. To be able to register physical activity, you first press that symbol.
-
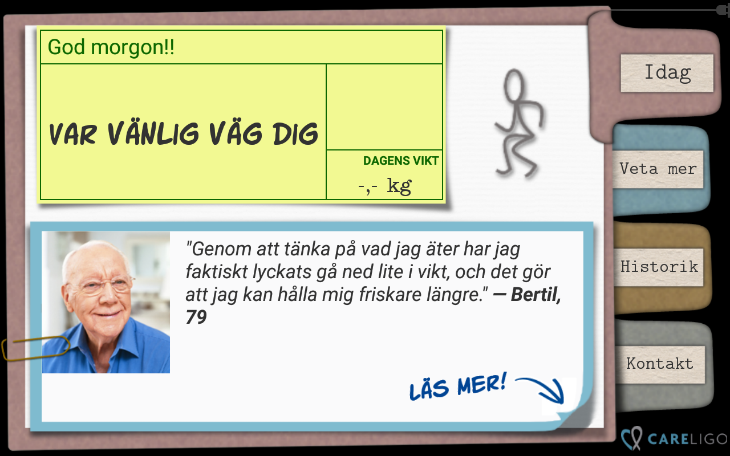
After the slide show, the appearance of the screen is back to normal, with the exception that the new symbol has appeared, and the user can now press that symbol to get to register physical activity.
- Once the symbol is pressed, this registration view appears.

Figure A5. Standard view after intro week. The stick figure is the icon that opens the registration view.

-
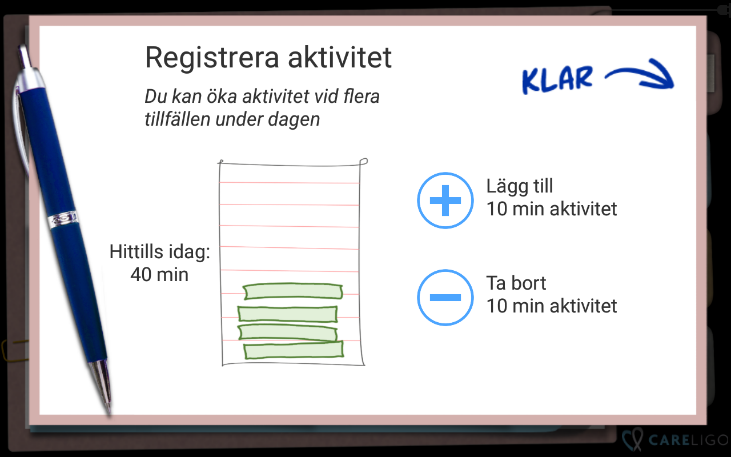
 This is the registration view.

Figure A6. The registration view.

- Physical activity is added or removed in increments of 10 minutes, by simply pressing the “+”-symbol or the “-“-symbol.

The physical activity added is shown graphically by “filling a jar” and by text, as a summarized total to the left (40 minutes in this picture). Activity can be added throughout the day as many times as you like.


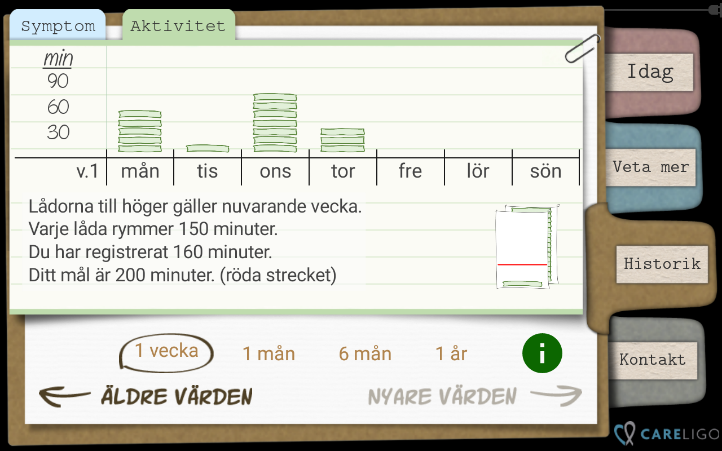


Figure A7. The History-tab, activity view.

- In the history (Swedish: ‘Historik’) tab, the user can view the registered activity the current week, with stacks of ten-minute increments on each day of the week. The total activity of the week is summarized graphically in one or more “jars” to the right, and in text to the left. If the user has set a goal for the current week, this is illustrated graphically and in text.
-
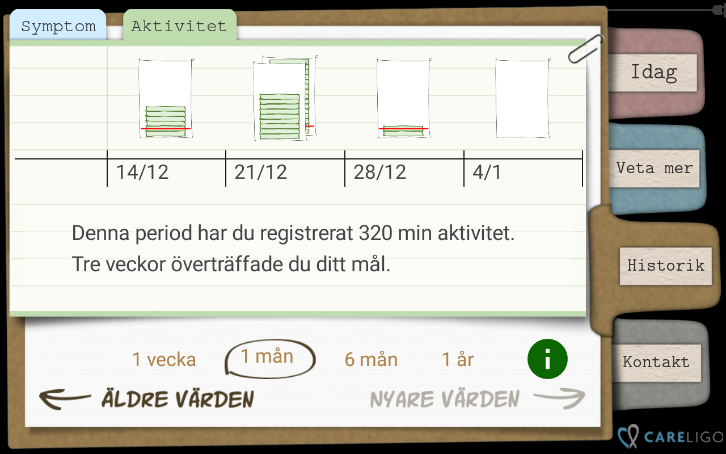
Using the zoom-options at the bottom, the user can zoom out and see the “jars” filled for each week.

Figure A8. Zoomed out view of previously registered physical activity.

-
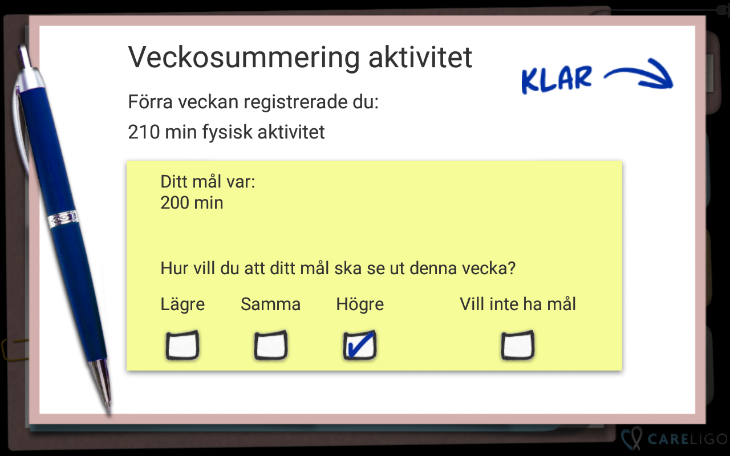
At the end of each week, the registered activity is summarized and compared to the set goal (if there was one). At this point the user can set the goal for next week.

Figure A9. Weekly summary and goal-setting view.

It is also possible to select to have no goal (Swedish: ‘Vill inte ha mål’). The option to set a goal will be available at each week-summary in this view.

A logical model was constructed to describe the intervention mechanistically and it is illustrated in Figure A3, based on principles from Kidger et al. (23). A way to visualise and present an understanding of the way an intervention is intended to work, the relationships between its constituents and the changes or results that is hoped to be achieved, is to construct a logical model (24). They are to illustrate the causal process through which an intervention leads to a desired outcome (25).


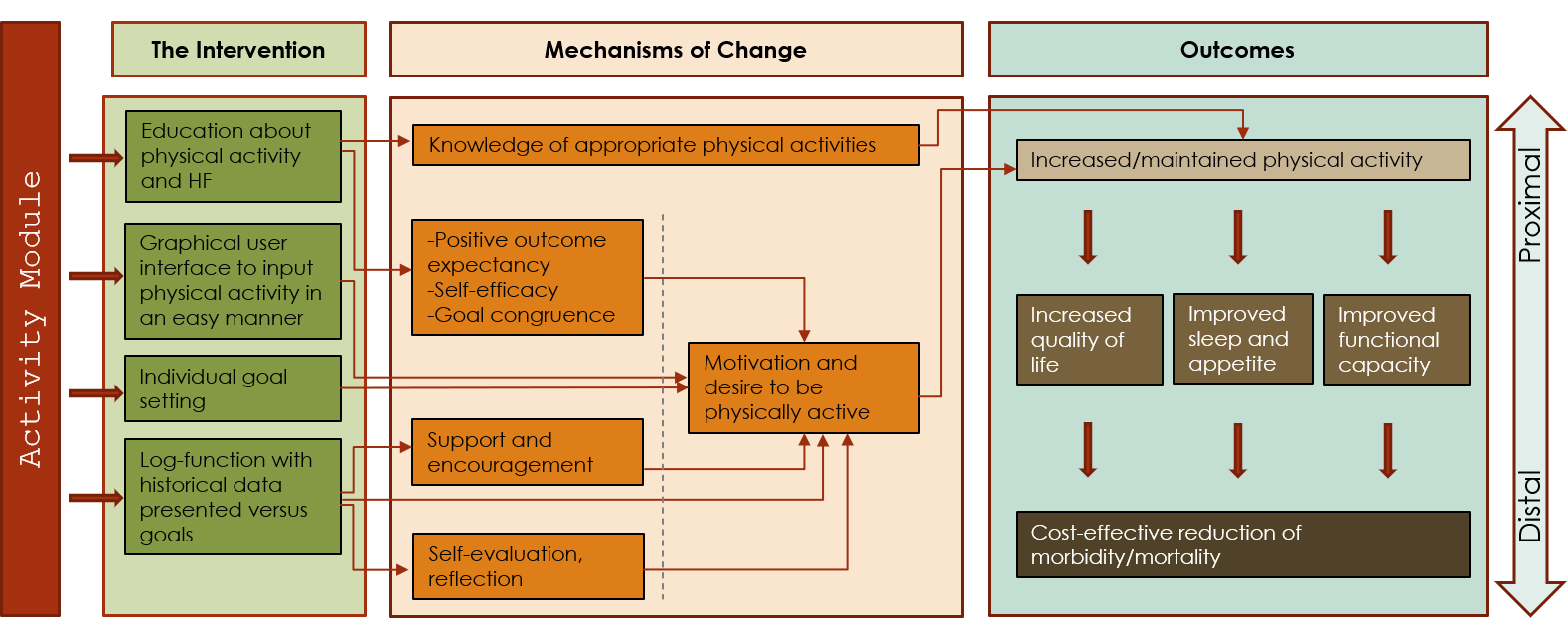


Figure A10. A logical model describing the intervention. The outcomes are hypotheses at this stage, based on literature reviews and interviews, and need to be verified.

The working prototype was now ready for a BVT to test the implementation in a real-life setting before it could be tested in the more formalized pilot.

### Build verification test

For the BVT, three elderly chronically ill people were recruited to use the activity coach prototype for two weeks. Their ages were 72, 75 and 68, two were male and one female. Everybody could use the activity coach as intended. There were some issues identified with the goal-setting functionality which need to be improved and there was also verbal feedback that despite the introduction, it was unclear to the users which activities actually qualify as physical activity. Three system crashes were found in the user-logs. Addressing these flaws in an updated version of the prototype concludes the implementation phase of the development process.

The “evaluation and refining phase” is described in the main article.

# References

1. Craig P, Dieppe P, Macintyre S, Michie S, Nazareth I, Petticrew MJB. Developing and evaluating complex interventions: the new Medical Research Council guidance. 2008;337:a1655.

2. O'Cathain A, Croot L, Duncan E, Rousseau N, Sworn K, Turner KM, Yardley L, Hoddinott P. Guidance on how to develop complex interventions to improve health and healthcare. BMJ open. 2019;9(8):e029954.

3. Wight D, Wimbush E, Jepson R, Doi L. Six steps in quality intervention development (6SQuID). Journal of Epidemiology and Community Health. 2016;70(5):520-5.

4. Ben-Zeev D, Schueller SM, Begale M, Duffecy J, Kane JM, Mohr DC. Strategies for mHealth research: lessons from 3 mobile intervention studies. Administration and Policy in Mental Health and Mental Health Services Research. 2015;42(2):157-67.

5. Chatzipavlou IA, Christoforidou SA, Vlachopoulou M. A recommended guideline for the development of mHealth Apps. Mhealth. 2016;2:21.

6. Whittaker R, Merry S, Dorey E, Maddison R. A development and evaluation process for mHealth interventions: examples from New Zealand. J Health Commun. 2012;17 Suppl 1(sup1):11-21.

7. Woods LS, Duff J, Roehrer E, Walker K, Cummings E. Patients’ Experiences of Using a Consumer mHealth App for Self-Management of Heart Failure: Mixed-Methods Study. JMIR human factors. 2019;6(2):e13009.

8. Jacobs MA, Graham AL. Iterative development and evaluation methods of mHealth behavior change interventions. J Current Opinion in Psychology. 2016;9:33-7.

9. Hawkins J, Madden K, Fletcher A, Midgley L, Grant A, Cox G, Moore L, Campbell R, Murphy S, Bonell C. Development of a framework for the co-production and prototyping of public health interventions. BMC public health. 2017;17(1):689.

10. Elo S, Kyngäs H. The qualitative content analysis process. Journal of Advanced Nursing. 2008;62(1):107-15.

11. Ryan P. Integrated Theory of Health Behavior Change: background and intervention development. Clin Nurse Spec. 2009;23(3):161-70; quiz 71-2.

12. Kwasnicka D, Dombrowski SU, White M, Sniehotta F. Theoretical explanations for maintenance of behaviour change: a systematic review of behaviour theories. Health Psychol Rev. 2016;10(3):277-96.

13. Voskuil VR, Robbins LB. Youth physical activity self-efficacy: a concept analysis. J Adv Nurs. 2015;71(9):2002-19.

14. Riegel B, Jaarsma T, Stromberg A. A middle-range theory of self-care of chronic illness. ANS Adv Nurs Sci. 2012;35(3):194-204.

15. Jaarsma T, Cameron J, Riegel B, Stromberg A. Factors Related to Self-Care in Heart Failure Patients According to the Middle-Range Theory of Self-Care of Chronic Illness: a Literature Update. Curr Heart Fail Rep. 2017;14(2):71-7.

16. Dickson VV, Tkacs N, Riegel B. Cognitive influences on self-care decision making in persons with heart failure. Am Heart J. 2007;154(3):424-31.

17. Franklin NC. Technology to promote and increase physical activity in heart failure. Heart failure clinics. 2015;11(1):173-82.

18. Beckers PJ, Denollet J, Possemiers NM, Wuyts K, Vrints CJ, Conraads VM. Maintaining physical fitness of patients with chronic heart failure: a randomized controlled trial. Eur J Cardiovasc Prev Rehabil. 2010;17(6):660-7.

19. Yehle KS, Plake KS. Self-efficacy and educational interventions in heart failure: a review of the literature. Journal of Cardiovascular Nursing. 2010;25(3):175-88.

20. Keessen P, Latour CHM, van Duijvenbode ICD, Visser B, Proosdij A, Reen D, Scholte op Reimer WJM. Factors related to fear of movement after acute cardiac hospitalization. BMC Cardiovascular Disorders. 2020;20(1):495.

21. Hovland-Tanneryd A, Melin M, Hagglund E, Hagerman I, Persson HE. From randomised controlled trial to real world implementation of a novel home-based heart failure tool: pooled and comparative analyses of two clinical controlled trials. Open heart. 2019;6(1):e000954.

22. Sahlin D, Rezanezad B, Edvinsson M, Bachus E, Melander O, Gerward S. Selfcare management intervention in heart failure (SMART-HF)-a multicentre randomized controlled trial. Journal of Cardiac Failure. 2021.

23. Kidger J, Evans R, Tilling K, Hollingworth W, Campbell R, Ford T, Murphy S, Araya R, Morris R, Kadir B. Protocol for a cluster randomised controlled trial of an intervention to improve the mental health support and training available to secondary school teachers–the WISE (Wellbeing in Secondary Education) study. BMC public health. 2016;16(1):1089.

24. Foundation WK. WK Kellogg Foundation logic model development guide: WK Kellogg Foundation; 2004.

25. Mills T, Lawton R, Sheard L. Advancing complexity science in healthcare research: the logic of logic models. BMC medical research methodology. 2019;19(1):55.
