# Supplementary material for: Usability and feasibility analysis of an mHealth-tool for supporting physical activity in people with heart failure
Source: BMC Med Inform Decis Mak. 2024 Feb 12;24:44. doi: 10.1186/s12911-024-02452-z (PMC10860324; doi:10.1186/s12911-024-02452-z)
Supplement: Supplementary file 2 — Appendix B – Interview Guide [file 12911_2024_2452_MOESM2_ESM.docx]

Appendix B

Interview guide

1. Describe your feelings and thoughts when you first saw the activity coach
2. Describe how those thoughts and feelings changed during the study
3. Tell me about your experiences using the activity coach
4. How has the activity coach affected you during these past four weeks?
5. What was good about the activity coach?
6. What was less good about the activity coach?
7. Do you experience any change in your physical activity after these weeks? (Motivation, amount of physical activity, knowledge, physical ability)
8. Has any aspect of how you think about, or relate to, physical activity changed during the course of the study? How?
9. What would you like to change about the activity coach? What features would you like to add?
10. Please share any other thoughts, ideas, or suggestions you have regarding the activity coach
